# Bimekizumab rescue therapy in a patient with relapsing polychondritis-associated aortitis

**DOI:** 10.1016/j.ero.2025.05.005

**Published:** 2025-06-11

**Authors:** Phillip Kremer, Simon Melderis, Nikolas Ruffer, Daniel Koehler, Konstanze Holl-Ulrich, Ina Kötter, Martin Krusche

**Affiliations:** 1 Department of Medicine III, University Medical Center Hamburg-Eppendorf, Martinistr. 52, 20246, Hamburg, Germany; 2 Department of Rheumatology and Clinical Immunology, Bad Bramstedt, Germany; 3 Department for Radiology and Nuclear Medicine, University Medical Center Hamburg-Eppendorf, Hamburg, Germany; 4 Pathology Hamburg, Medizinisches Labor Nord MVZ GmbH, Hamburg, Germany

## Abstract

Relapsing polychondritis (RP) is a rare inflammatory disease characterised by recurrent inflammation of cartilaginous structures, especially of the ears, nose and respiratory tract, with a broad spectrum of systemic features. Furthermore, cardiovascular involvement is a rare but potentially life-threatening complication.

We report the case of a 28-year-old woman with RP and refractory disease activity complicated by an aortic aneurysm, severe aortic regurgitation and embolic strokes. Positron emission tomography/computed tomography (PET/CT) imaging confirmed large vessel vasculitis of the aorta. Despite multiple immunosuppressive therapies, including glucocorticoids, methotrexate, cyclophosphamide and biologics targeting tumour necrosis factor-alpha (TNF-α), interleukin (IL)-1 and IL-6, high disease activity persisted. Rescue therapy with bimekizumab, a dual inhibitor of IL-17A and IL-17F, was associated with the normalisation of inflammation markers, PET/CT features and sustained clinical remission.

To the best of our knowledge, this is the first report of successful treatment of RP-associated aortitis with bimekizumab, highlighting the potential role of IL-17A and IL17F in RP pathogenesis. These findings provide a foundation for further exploration of IL-17 inhibition in RP-associated vasculitis.

Dear Editor,

Relapsing polychondritis (RP) is a rare inflammatory disease characterised by recurrent inflammation of cartilaginous structures and additional systemic features [1]. Here, we report the case of a 28-year-old woman who was diagnosed with RP, affecting the cartilaginous structures of ears and nose (causing ‘saddle-nose’-deformity, Fig, B), and arthritis in 2020 (Fig, timepoint 0). Conventional immunosuppression including glucocorticoids, methotrexate and adalimumab failed to control disease activity. Furthermore, the patient developed a large aortic root and arch aneurysm (Fig, D) with severe aortic regurgitation (at 10 months) requiring emergency surgical ascending aortic replacement (Hemi-Yacoub procedure, 26-mm prosthesis). Histopathologic evaluation of the resected specimen confirmed large vessel vasculitis (Supplementary Figs S1 and S2). Subsequent [Flourine-18] fluorodeoxyglucose positron emission tomography/computed tomography ([^18^F]FDG PET/CT) imaging (at 18 months) also revealed persistent disease activity of the entire aortic wall. The disease course was further complicated by multiple, bilateral embolic strokes and a left-sided renal infarction (at 18 months). Further immunosuppressive therapies (from 18 to 38 months) including high-dose glucocorticoids, cyclophosphamide, tocilizumab, abatacept, anakinra and infliximab failed to control disease activity (Fig, A,C). Persistent aortitis caused an additional 6 cm thoracic aortic aneurysm (at 27 months, Fig, D), requiring repeat surgery with initially biological and later on mechanical aortic valve replacement (Edwards Inspiris 27 mm) as well as full aortic reconstruction using vascular prostheses (22/24/150-mm Thoraxflex® prosthesis, TEVAR, of the descending aorta). Based on the results from the TitAIN-trial [2] on the efficacy of interleukin (IL)-17 inhibition in large vessel vasculitis, secukinumab with concomitant methotrexate therapy was introduced. Secukinumab therapy resulted in a partial response with a decrease in serological inflammation. However, clinical and serological inflammation persisted and positron emission tomography/computed tomography (PET/CT) imaging during secukinumab therapy showed ongoing large vessel inflammation in the thoracic aorta (Fig, A,E). Therefore, rescue therapy with bimekizumab and leflunomide was initiated. This resulted in a sustained absence of clinical, laboratory and radiographic (Fig, F) inflammation over a follow-up period of 12 months. Furthermore, prednisolone could be tapered to a daily dose of 4 mg.

**Figure:**
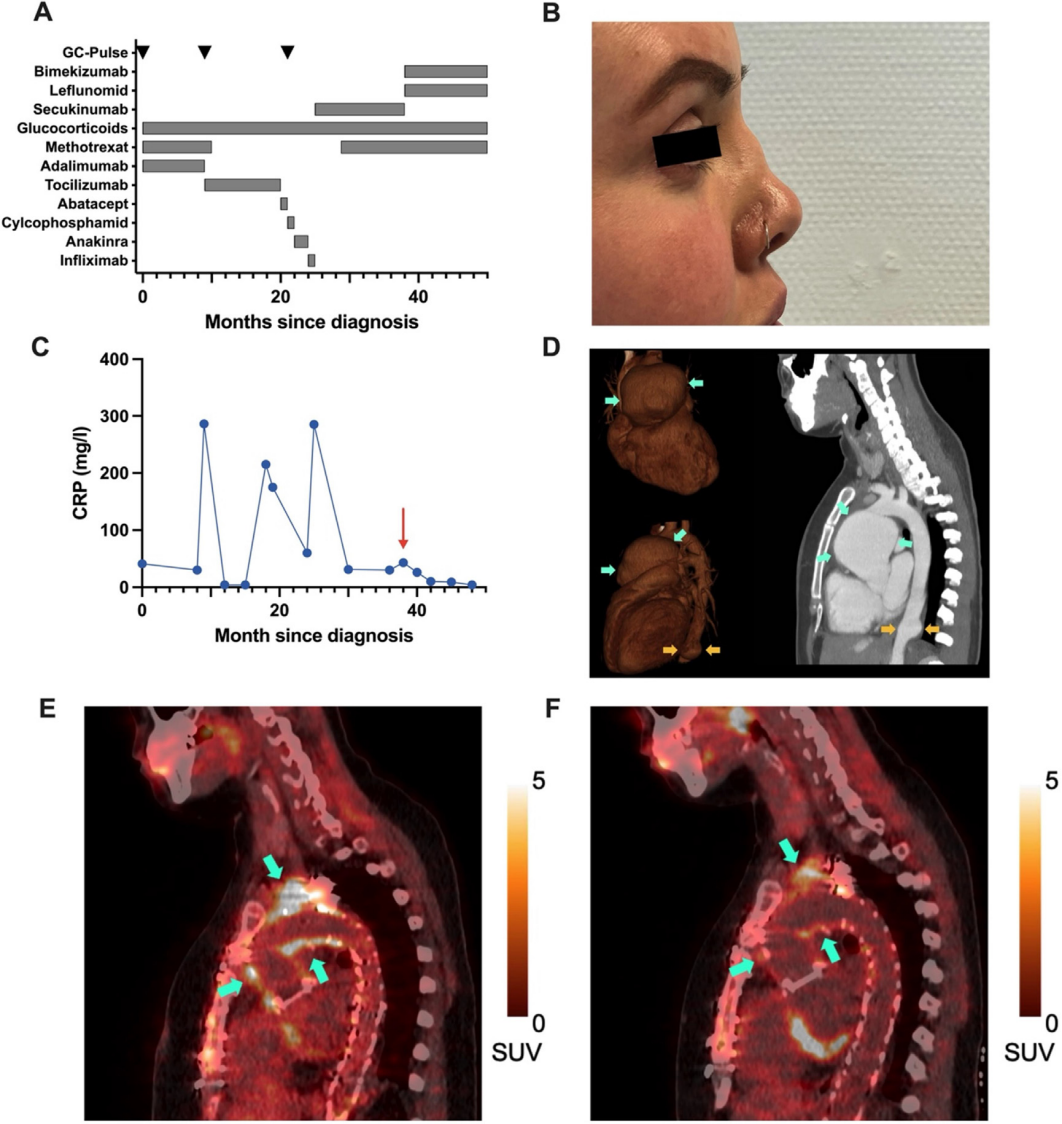
(A) Sequential immunotherapy during the clinical course. (B) ‘Saddle-nose’ deformity due to severe destruction of nasal cartilage. (C) C-reactive protein (CRP) level during the clinical course, the arrow indicates time at bimekizumab initiation. (D) Three-dimensional reconstruction of the heart and large intrathoracic vessels in frontal (upper left) and left lateral (lower left) view based on contrast-enhanced computed tomography of the thorax (portal venous phase). ‘Maximum intensity projection’ of the same image (right) in parasagittal slice guidance (5-mm slice thickness, 1-mm slice distance). Image of an aneurysm of the ascending thoracic aorta measuring max. of 8.5 cm (blue arrows) and an aneurysm of the descending thoracic aorta measuring max. of 2.7 cm (yellow arrows). E, F, 18F-fluorodeoxyglucose ([18F]FDG) positon emission tomography-computed tomograühy PET/CT images showing FDG-uptake before (at month 33, during secukinumab therapy) (E) and after (at month 48) (F) treatment with bimekizumab, with a significant decrease in metabolic activity in the thoracic aorta. SUV = standardized uptake value.

During the entire disease course, regular monitoring for infectious complications (tuberculosis, hepatitis, vaccination status) was performed. No adverse events or infections were observed, and no infections were reported, including invasive fungal infections like candidiasis. However, given the patient’s history of multiple prior therapies and surgical interventions, it remains uncertain whether remission can be attributed solely to bimekizumab. This is particularly relevant as the treatment regimen included a combination of bimekizumab, leflunomide, prednisolone and methotrexate.

Cardiovascular involvement including aortitis represents a rare but potentially life-threatening complication of RP that requires interdisciplinary evaluation, including surgical approaches. Current therapeutic considerations are largely based on anecdotal evidence and controlled studies are lacking [3]. Surgical treatment of aneurysmal vascular changes represents an important component of therapy in addition to immunosuppressive treatment. Ideally, complete disease remission should be achieved before surgical intervention because recurrence of inflammatory aneurysms despite surgery has been reported [4].

To the best of our knowledge, this is the first report of successful treatment of RP-associated aortitis with bimekizumab, a humanised monoclonal antibody targeting IL-17A and IL-17F. Recently, secukinumab proved to be safe and effective in a proof-of-concept phase 2 study (TitAIN-trial), which supports the use of IL-17 inhibition in giant cell arteritis, as the prime example of large vessel vasculitis [2]. Moreover, a recent study by Tian et al [5] underlines the efficacy of secukinumab therapy in patients with Takayasu arteritis. As a potent proinflammatory cytokine, IL-17 plays a pivotal role in multiple inflammatory processes and is constantly regulated by the host’s health status [6]. Findings from multiple studies highlight the role of IL-17, in particular IL-17A and IL-17F, in vascular inflammation, coagulation and thrombosis, thus driving vasculitis and aneurysm formation [6]. By forming heterodimers, IL-17A and IL-17F exert a synergistic effect, leading to a stronger proinflammatory response. Limited preclinical data based on a cytokine study may suggest the upregulation of IL-17 in RP [7].

Our results could lead to the hypothesis that combined inhibition of IL-17A and IL-17F might be more effective in suppressing the inflammation of RP. Thus, bimekizumab appears to be a safe and effective treatment option for this disease.

## CRediT authorship contribution statement

**Phillip Kremer:** Writing – original draft, Visualization, Software, Methodology, Formal analysis. **Simon Melderis:** Writing – review & editing. **Nikolas Ruffer:** Writing – review & editing. **Daniel Koehler:** Writing – review & editing, Data curation. **Konstanze Holl-Ulrich:** Writing – review & editing, Data curation. **Ina Kötter:** Writing – review & editing, Supervision. **Martin Krusche:** Writing – review & editing, Supervision, Project administration, Conceptualization.

## Competing interests

PK has received speaker honoraria from AlphaSigma. IK has received speaker and consultancy honoraria from GSK, Eusapharm, Boehringer, Abbvie, Janssen, Lilly, Medac, Novartis, and Sobi. MK has received speaker and consultancy honoraria from Novartis, Sobi, Abbvie, Roche/Chugai, and UCB. All other authors report no conflicts of interest.

## Appendix Supplementary materials

Image, application 1
