# Supplementary material for: Bimekizumab rescue therapy in a patient with relapsing polychondritis-associated aortitis
Source: EULAR Rheumatol Open. 2025 Jun 11;1(2):60–2. doi: 10.1016/j.ero.2025.05.005 (PMC13292415; doi:10.1016/j.ero.2025.05.005)

**Supplemental data:**

**Supplementary Figure S1:** A and B: Histopathological section of aorta (A: H&E, B: Elastica-van-Gieson). Band-like smooth muscle cell loss (arrowheads), dense mixed inflammatory infiltrate of narrowed and disrupted media (red asterisk), fragmentation and loss of elastic fibers (arrows). C and D: Mixed inflammatory infiltrate of aortic wall, C: macrophages (CD163), D: T-lymphocytes (CD3), and neutrophils (not shown).

**
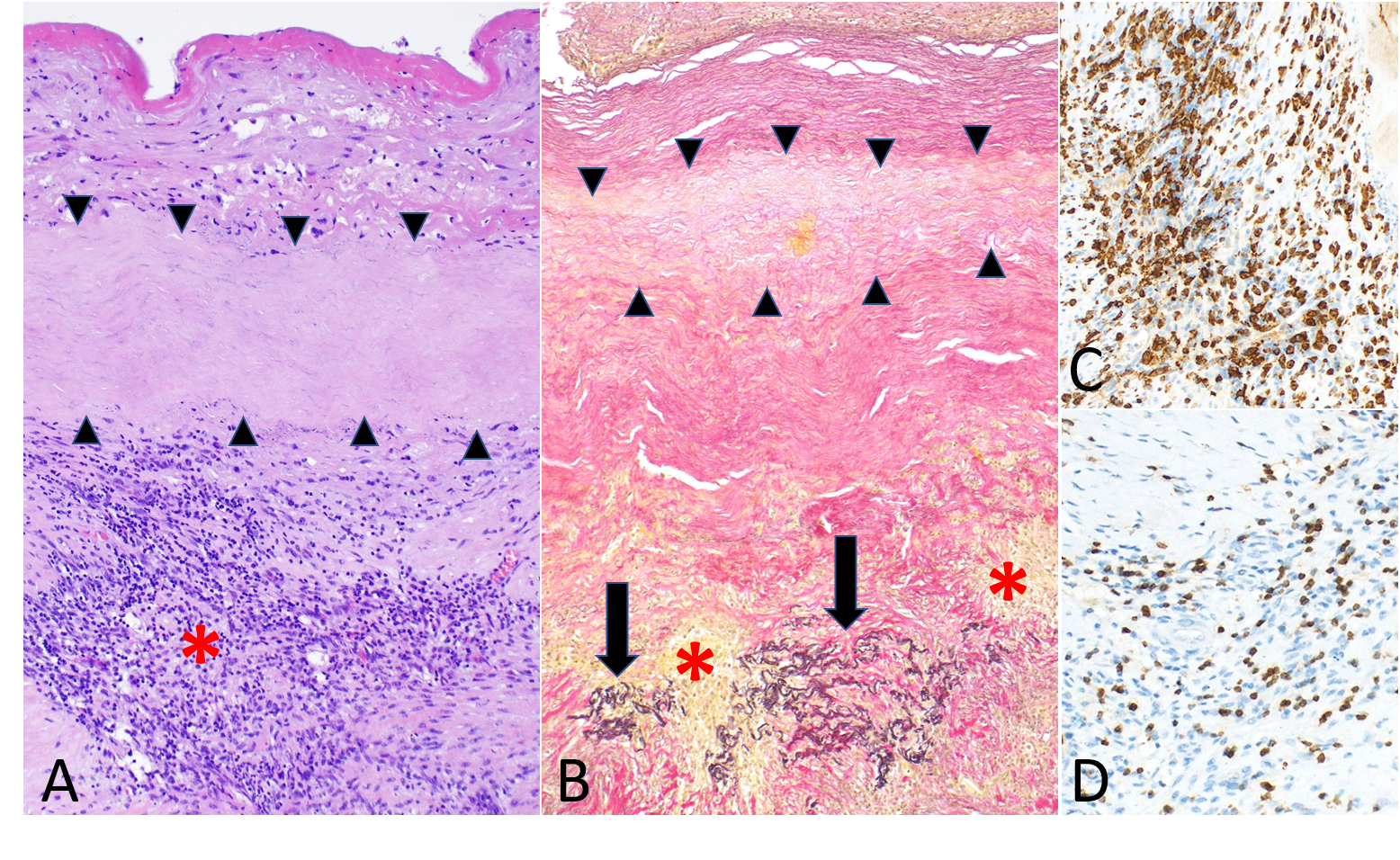
**

**Supplementary Figure S2: Dosage of immunosuppressive drugs used during the disease course.**
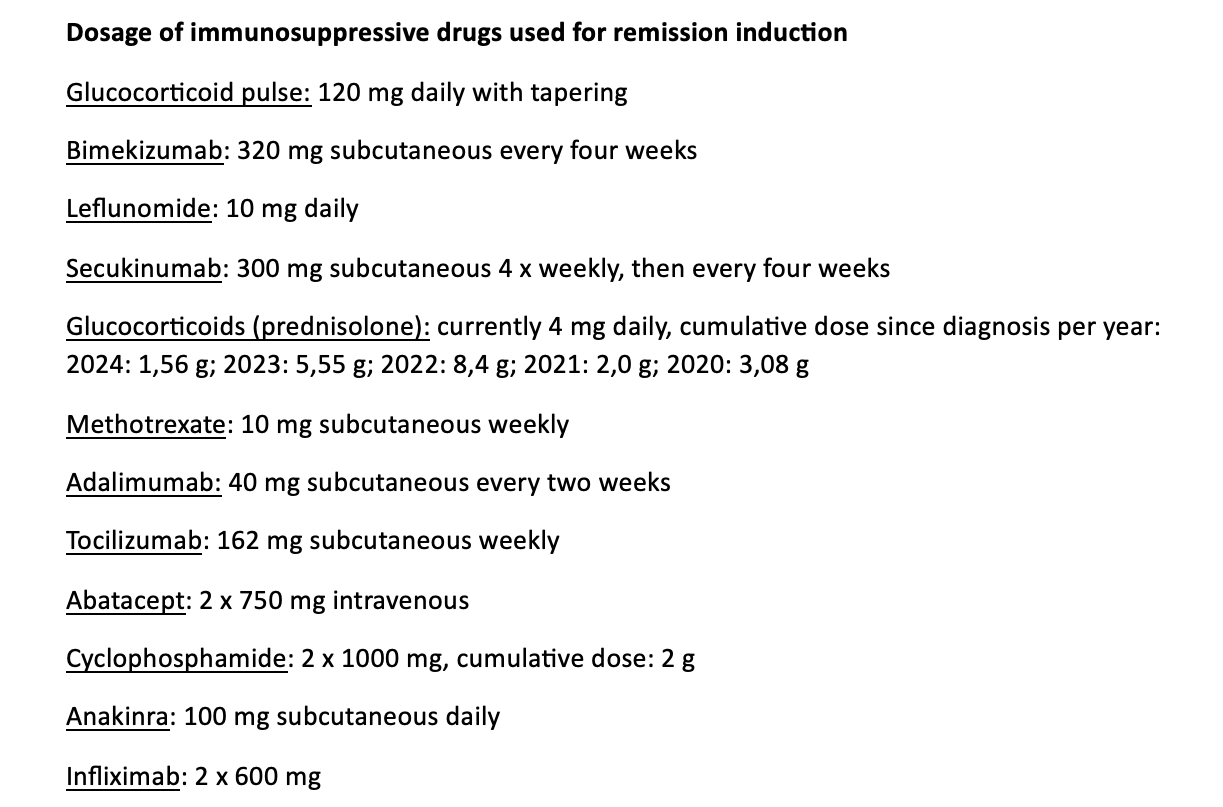

Supplement: Supplementary file 1 [file mmc1.docx]
